# Development of activated endothelial targeted high-density lipoprotein nanoparticles

**DOI:** 10.3389/fphar.2022.902269

**Published:** 2022-08-29

**Authors:** Minzhi Yu, Kristen Hong, Reheman Adili, Ling Mei, Lisha Liu, Hongliang He, Yanhong Guo, Y. Eugene Chen, Michael Holinstat, Anna Schwendeman

**Affiliations:** ^1^ Department of Pharmaceutical Sciences and the Biointerfaces Institute, University of Michigan, Ann Arbor, MI, United States; ^2^ Department of Pharmacology, University of Michigan Medical School, Ann Arbor, MI, United States; ^3^ Department of Pharmaceutics, School of Pharmacy, China Pharmaceutical University, Nanjing, China; ^4^ State Key Laboratory of Bioelectronics, Jiangsu Key Laboratory for Biomaterials and Devices, School of Biological Science and Medical Engineering, Southeast University, Nanjing, China; ^5^ Department of Internal Medicine, Frankel Cardiovascular Center, University of Michigan, Ann Arbor, MI, United States

**Keywords:** VCAM-1, endothelium, inflammation, anti-inflammation, high-density lipoprotein

## Abstract

Endothelial inflammation is an important pathophysiological driving force in various acute and chronic inflammatory diseases. High-density lipoproteins (HDLs) play critical roles in regulating endothelial functions and resolving endothelial inflammation. In the present study, we developed synthetic HDLs (sHDLs) which actively target inflamed endothelium through conjugating vascular cell adhesion protein 1 (VCAM-1) specific VHPK peptide. The active targeting of VHPK-sHDLs was confirmed *in vitro* on TNF-α activated endothelial cells. VHPK-sHDLs presented potent anti-inflammatory efficacies *in vitro* through the reduction of proinflammatory cytokine production and inhibition of leukocyte adhesion to activated endothelium. VHPK-sHDLs showed increased binding on inflamed vessels and alleviated LPS-induced lung inflammation *in vivo*. The activated endothelium-targeted sHDLs may be further optimized to resolve endothelial inflammation in various inflammatory diseases.

## Introduction

Inflammation is a common denominator in the pathophysiology of a broad array of diseases, including atherosclerosis, sepsis, and autoimmune diseases (Wang et al., 2015; Hotchkiss et al., 2016; Kobiyama and Ley, 2018). The vascular endothelium plays important roles in the initiation and progression of inflammation (Murdaca et al., 2012; Gimbrone and Garcia-Cardena, 2016; Grandl and Wolfrum, 2018; Dolmatova et al., 2021). Various stimuli in inflammatory diseases, such as endotoxins, mechanical stress, oxidative stress, and circulating proinflammatory cytokines, could convert endothelial cells from a resting state to an activated state (Peters et al., 2003; Zhang, 2008; Chistiakov et al., 2017; Incalza et al., 2018). Activated endothelial cells produce proinflammatory cytokines and chemokines, recruiting leukocytes such as monocytes and neutrophils to the site of inflammation (Theofilis et al., 2021). At the same time, activated endothelial cells express adhesion molecules, including selectins, intercellular adhesion molecule-1 (ICAM-1), and vascular cell adhesion molecule-1 (VCAM-1), which enables cellular adhesion and migration of recruited immune cells (Leeuwenberg et al., 1992; Collins et al., 1993). In the case of inflammatory diseases, the inflammation often remains unresolved due to dysregulated inflammatory responses. The unresolved inflammation creates a vicious cycle of persisting endothelial activation, recruitment of immune cells, inflammatory responses, and tissue damage (Khatami, 2011; Viola and Soehnlein, 2015). Breaking such a cycle by alleviating endothelial activation and inflammation would be a potential treatment strategy for inflammatory diseases (Buechler et al., 2017; Back et al., 2019).

High-density lipoproteins (HDLs) are a group of lipoproteins mainly composed of esterified and free cholesterol, phospholipids, and apolipoproteins which predominately involve ApoA-1 and ApoE (Kontush et al., 2015). As a major player in maintaining cholesterol homeostasis, HDLs induce cholesterol efflux from peripheral cells by interacting with various receptors on cell membranes such as ATP-binding cassette transporter A1 (ABCA-1), ATP-binding cassette transporter G1 (ABCG-1), and scavenger receptor BI (SR-BI). HDLs also play crucial roles in regulating inflammation responses and endothelial functions (Kajani et al., 2018), which can be dependent or independent of the cholesterol mobilization effects of HDLs (Prosser et al., 2012; Bonacina et al., 2019). For example, it was found that through ABCA-1 mediated efflux, HDLs modulate the cholesterol content of lipid rafts, inhibiting the trafficking of Toll-like receptor 4 (TLR-4) to cellular membranes and inhibiting the activation of downstream inflammation pathways (Zhu et al., 2010). The lipid raft disruption caused by cholesterol efflux could also inhibit the translocation of NADPH oxidase 4 (NOX-4), inhibiting the generation of reactive oxygen species (ROS) (Umemoto et al., 2013). Through binding with SR-BI, HDLs activate the phosphatidylinositol-3-kinase-protein-kinase-B (PI3K-AKT) pathway and mitogen-activated protein kinase (MAPK) pathway. Such kinase cascade activation stimulates endothelial nitric oxide synthase (eNOS) and increases nitric oxide (NO) production (Shaul, 2002; Mineo et al., 2006). In terms of cholesterol efflux independent anti-inflammatory mechanisms, HDLs could effectively neutralize lipopolysaccharide (LPS) and lipoteichoic acid (LTA) during bacteria infection, reducing LPS- and LTA-induced inflammatory responses (Morin et al., 2015). Overall, HDLs have been shown to reduce the expression of adhesion molecules, alleviate intracellular oxidative stress, prevent endothelial cell apoptosis, and inhibit the secretion of proinflammatory cytokines on activated endothelial cells (Cockerill et al., 1995; Sugano et al., 2000; de Souza et al., 2010). Such anti-inflammation and endothelial protective functions of HDL make it an appealing treatment option for inflammatory diseases.

Inspired by endogenous HDLs, various synthetic HDLs (sHDLs) composed of lipids and ApoA-1 or ApoA-1 mimetic peptides have been developed (Nankar et al., 2022). Several sHDL candidates, such as CSL112, CER-001, ETC 642, have entered clinical trials (Khan et al., 2003; Nicholls et al., 2018; Gibson et al., 2021) and showed favorable safety profiles. It is worth noting that the current sHDL therapies were originally designed for atherosclerosis treatment (Zakiev et al., 2017; Abudukeremu et al., 2021). As a result, the formulation development was mainly focused on optimizing the reverse cholesterol transport capacities of sHDLs to reduce atheroma plaques. However, in recent years, there has been a growing interest in broadening the therapeutic applications of sHDL products to other inflammatory diseases, including sepsis, COVID-19, and autoimmune diseases (Kim et al., 2020; Guo et al., 2022; Tanaka et al., 2022). Thus, additional focus has been put on optimizing the anti-inflammatory and endothelial protective functions of sHDLs.

**SCHEME 1:**
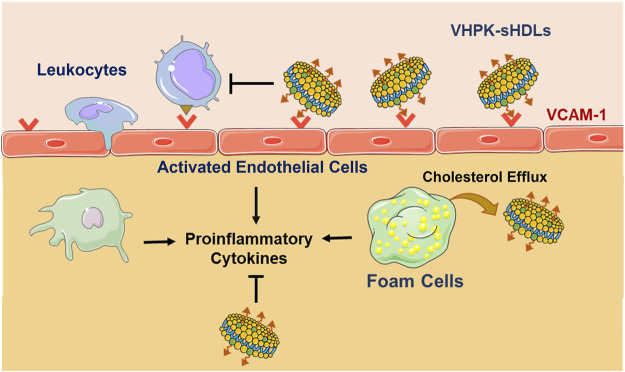
Schematic illustration of VCAM-1 mediated active targeting to inflamed endothelium and anti-inflammatory mechanisms of VHPK-sHDLs. The figure is created with materials from Servier Medical Art.

Among the variety of adhesion molecules on inflamed endothelium, VCAM-1 has recently received much research interest as a biomarker and targeting site for inflamed endothelium. Expressed in inflamed endothelium, VCAM-1 enables cell adhesion through binding with very late antigen-4 (VLA-4) expressed on the surface of leukocytes and lymphocytes (Alon et al., 1995). One VLA-4 mimicking peptide, VHPKQHR, was found to have high binding efficiency with VCAM-1 in phage display studies (Kelly et al., 2006). Various peptides with the VHPKQHR motif (VHPK peptides) have since been widely used in developing inflamed endothelial targeting imaging agents and drug delivery systems (Ailuno et al., 2020). In the present study, a VHPK peptide is conjugated to sHDLs. It is hypothesized that the conjugation of the targeting peptide would enable an active targeting of sHDLs to activated endothelial cells, increasing the distribution of sHDLs on inflamed endothelium and enabling stronger inflammation resolution effects (Scheme 1). To test this hypothesis, the endothelial targeting efficiency and anti-inflammatory effects of VHPK-sHDLs were examined *in vitro.* The *in vivo* targeting effect of VHPK-sHDLs to inflamed endothelium was further examined using intravital microscopy. As a proof-of-concept experiment, an LPS-induced lung inflammation model was used to evaluate the anti-inflammatory effects of VHPK-sHDLs *in vivo*.

## Materials and methods

### Materials

ApoA-1 mimetic 22A peptide (PVLDLFRELLNELLEALKQKLK) was synthesized by Genscript Inc. (Piscataway, NJ). VHPK peptide (VHPKQHRGGSKGC) and scrambled peptide (QRHPHVKGGSKGC) were synthesized by RS Synthesis LLC (Louisville, KY) and GeneMed Biotechnologies Inc. (South San Francisco, CA). 1,2-dimyristoyl-sn-glycero-3-phosphocholine (DMPC) was purchased from NOF America Corporation (White Plains, NY). 1,2-dioleoyl-sn-glycero-3-phosphoethanolamine-N-[3-(2-pyridyldithio)propionate] (DOPE-PDP) was purchased from Avanti Polar Lipids (Alabaster, AL). LPS from *Escherichia coli* O111 (L2630):B4 was purchased from Sigma. Recombinant human TNF-α and mouse VCAM-1/CD106 antibody (AF643) were purchased from R&D Systems (Minneapolis, MN). Human VCAM-1 antibody (ab134047) was purchased from Abcam (Waltham, MA). 1,1′-dioctadecyl-3,3,3′,3′- tetramethylindodicarbocyanine (DiD) and 3,3′-dioctadecyloxacarbocyanine perchlorate (DiO) were obtained from Thermo Fisher Scientific (Waltham, MA). Mouse IL-6, mouse MCP-1, human IL-6, human IL-8, human IL-1β, and human TNF-α ELISA kits were purchased from Invitrogen (Waltham, MA).

### Cell culture

Human umbilical vein endothelial cells (HUVEC) from pooled donors were purchased from Lonza (Cat #: C2519A, Morristown, NJ). Cells were cultured in EGM-2 complete media (Lonza). HUVEC cells were between passages three to seven for all experiments. THP-1 cells were obtained from the American Type Culture Collection (ATCC) (Cat# TIB-202) and kept in RPMI-1640 media supplemented with 10% fetal bovine serum (FBS), 1% Penicillin-Streptomycin (10,000 U/mL), and 0.05 mM 2-mercaptoethanol. J774. A1 cells were cultured in DMEM media supplemented with 10% fetal bovine serum (FBS) and 1% Penicillin-Streptomycin (10,000 U/mL). All cells were cultured in a 37°C incubator with 5% CO_2_.

### Synthesis of peptide-DOPE conjugates

Peptide-DOPE conjugates were synthesized as described previously with slight modification (Kuai et al., 2017). Briefly, VHPK or scrambled peptide was reacted with DOPE-PDP (peptide:DOPE-PDP n:n = 1.5:1) in anhydrous DMSO for 12 h. To determine the conjugation efficiency, the unreacted DOPE-PDP content at the end of the reaction was quantified on an ultra-performance liquid chromatography-mass spectrometry (UPLC-MS) with a hydrophilic interaction liquid chromatography (HILIC) column. The mobile phase consisted of (A) water with 0.1% formic acid (FA), (B) acetonitrile with 0.1% FA, (C) methanol with 0.1% FA, and (D) 100 mM ammonium formate water solution. The flow rate was 0.4 ml/min. The samples were eluted in a gradient with 47.5% B, 47.5% C, and 5% D as initial conditions, which changed to 17% A, 17% B, 39% C, and 5% D in 2 min. DOPE-PDP was detected in a positive mode at m/z = 941.81. The conjugation percentage was calculated by unreacted DOPE-PDP concentrations in the reaction mixture before and after conjugation (Supplementary Figure S2A).

### Preparation and characterization of VHPK-sHDLs

The unconjugated, non-targeted sHDLs (NT-sHDL) were prepared by the lyophilization-rehydration method. DMPC and 22A were dissolved and mixed in acetic acid in a weight ratio of 2:1, followed by lyophilization. The lyophilized powder was then rehydrated by PBS (pH 7.4), followed by three thermocycles with 5 min incubation at 37°C and 5 min ice bath for each cycle. The purity of the prepared sHDL was analyzed on a Tosoh TSK gel G3000SWxl column with a PBS flow rate of 1 ml/min and UV detection at 220 nm. For fluorescently labeled sHDLs, DiD or DiO was added to DMPC and 22A mixture prior to lyophilization.

The VHPK-DOPE conjugate (VHPK-DOPE) or scrambled peptide-DOPE conjugate (Scr-DOPE) were then added to NT-sHDLs with a DOPE:total lipid molar ratio of 1:20, followed by 2 h incubation at room temperature under shaking. The resulting VHPK peptide conjugated sHDL (VHPK-sHDL) or scrambled peptide conjugated sHDL (Scr-sHDL) were purified by 7k MWCO Zeba™ spin desalting columns (Thermo Scientific, Waltham, MA). To determine the percentage of VHPK-DOPE inserted to sHDLs, VHPK-DOPE contents before and after desalting were quantified by UPLC-MS with an ACQUITY UPLC BEH300 C4 column. The mobile phase consisted of (A) water with 0.1% FA, (B) acetonitrile with 0.1% FA, and (C) methanol with 0.1% FA. The flow rate was 0.3 ml/min. The samples were analyzed with a gradient elution of A:B:C of 85:10:5 to 7:62:31 during 0–7 min, followed by an isocratic elution during 7–9 min, and a gradient of A:B:C of 7:62:31 to 85:10:5 during 9–11 min. VHPK-DOPE was detected in a positive mode at m/z = 741.3. The insertion percentage was calculated by dividing VHPK-DOPE content after the desalting process by the total amount of VHPK-DOPE before desalting (Supplementary Figure S2B).

The particle size and zeta potential of different sHDLs were determined by dynamic light scattering (DLS) on a Malvern Zetasizer Nano ZSP (Westborough, MA). The particle size distribution was measured in PBS with a 22A concentration of 1 mg/ml. Zeta potential was measured with a 22A concentration of 0.1 mg/ml in 10 mM phosphate buffer. To assess the morphology of different sHDLs, samples were loaded on a carbon film-coated 400 mesh copper grid from Electron Microscopy Sciences (Hatfield, PA), followed by negatively stained with 1% (w/v) uranyl formate and dried. The samples were imaged with 100 kV Morgagni transmission electron microscopy (TEM) with a Gatan Orius CCD.

### Cytotoxicity evaluation

HUVEC cells were seeded to 96-well plates at a density of 1 × 10^4^ cells/well and cultured overnight. Cells were incubated with different sHDLs at indicated 22A concentrations for 24 h. The cell viability was then determined using CellTiter 96^®^ AQueous One Solution Cell Proliferation Assay according to the protocol provided by the manufacturer.

### Cellular binding assay

HUVEC cells were seeded to 12-well plates and cultured to reach confluence before experiments. Endothelial inflammation was induced by pretreatment of TNF-α at 2 ng/ml for 8 h. Cells were fixed with 2% paraformaldehyde (PFA) for 15 min, followed by incubation with DiD-labeled sHDLs at a 22A concentration of 5 μg/ml for 30 min. The fluorescent intensity was determined by flow cytometry. For confocal microscopy imaging, HUVECs were seeded to 4-well chamber slides and cultured to reach confluence. The cells were fixed with 2% PFA at 4°C for 30 min, washed with PBS, and incubated with DiD-labeled sHDLs (22A concentration 5 μg/ml) at 37°C for 15 min. For VCAM-1 blocking, cells were pre-incubated with human VCAM-1 antibody at 10 μg/ml for 30 min before incubation of sHDLs. The slides were then washed with PBS, mounted using DAPI-containing mounting media, and imaged using a confocal microscope.

### Cholesterol efflux assay

J774. A1 cells were seeded in 24-well plates at a density of 2.5 × 10^5^ cells/well and incubated overnight. Cells were then labeled overnight with 1 μCi/ml [^3^H] cholesterol in DMEM containing 0.3% fatty acid-free bovine serum albumin (BSA) and 5 μg/ml ACAT inhibitor Sandoz 58-035. Cells were then washed with PBS twice and incubated in DMEM containing 0.3% BSA and 5 μg/ml ACAT inhibitor Sandoz 58-035 for 24 h. After being washed with PBS, cells were incubated with different sHDLs for 4 h at 22A concentrations of 5, 10, 25, or 50 μg/ml in DMEM containing 0.3% BSA. At the end of incubation, media was collected, and cells were lysed with 0.1% SDS in 0.1 M NaOH. Radioactive counts in media and cell lysis fractions were measured by liquid scintillation counting using Perkin Elmer Tri-Carb 2910 TR (Waltham, MA). The cholesterol efflux percentage was calculated by dividing the media count by the sum of the media and cell counts.

### Anti-inflammatory study

For anti-inflammatory studies on THP-1 derived macrophages, THP-1 cells were seeded to 24-well plates at a density of 2 × 10^5^/well. THP-1 cells were incubated with 50 ng/ml phorbol 12-myristate 13-acetate (PMA, Sigma) for 48 h to induce macrophage differentiation. The differentiated, adherent cells were washed with PBS and were allowed to rest in PMA-free media for 24 h. THP-1 derived macrophages were then co-incubated with 100 ng/ml LPS and sHDLs at a 22A concentration of 10, 20, or 50 μg/ml for 12 h. For anti-inflammatory studies on HUVEC cells, HUVEC cells were seeded to 24-well plates at a density of 5 × 10^4^/well. After overnight incubation, HUVEC cells were co-incubated with 100 ng/ml LPS and sHDLs at a 22A concentration of 10, 20, or 50 μg/ml for 12 h. At the end of incubation, the cell culture media was collected, and the cytokine concentrations were quantified by ELISA.

### Monocyte adhesion assay

HUVEC cells were seeded to 35 mm glass-bottom dishes and cultured until confluent. Cells were treated with 2 ng/ml TNF-α for 16 h to induce inflammatory responses. Cells without TNF-α pretreatment were used as control cells. THP-1 cells were fluorescently labeled by incubating cells with 0.5 µM BCECF-AM in PBS for 30 min. HUVEC cells were then treated with different sHDLs (22A 100 μg/ml) for 1.5 h. Fluorescently labeled THP-1 cells (2 × 10^5^) were subsequently added to each well. After 0.5 h incubation, the media containing unbound THP-1 cells was discarded. Cells were gently washed with PBS 3 times. Then cells were fixed with 2% PFA, sealed with coverslips, and visualized by confocal microscopy. The numbers of bound THP-1 in five random fields were counted for each dish.

### Intravital microscope

All animal experiments in the present study were approved by the Institutional Animal Care and Use Committee (IACUC) of the University of Michigan. C57BL/6 mice aged 3–4 weeks were obtained from Charles River Breeding Laboratories (Portage, MI). To induce endothelium inflammation, mice were pretreated with LPS (10 mg/kg i. p.) 3 h before sHDL administration. Mice without any treatment were used as the control group. Before imaging, mice were anesthetized with ketamine/xylazine mixture (Ketamine, 100 mg/kg; xylazine, 10 mg/kg) i. p. and placed on a heated stage. The mesentery was carefully exposed on a glass coverslip through a midline laparotomy. The mouse was then positioned on the microscopic stage, and blood flow in mesenteric venules was imaged using Zeiss Axio Observer Z1 Marianas Microscope. DiO-labeled NT-sHDL or VHPK-sHDL was injected i. v. at a 22A dose of 5 mg/kg. The DiO fluorescent signal of the mesentery was imaged at 10-, 30-, and 90-min post-injection of sHDL. The exposure time was kept as 200 ms throughout all imaging. The average fluorescent intensity was quantified by ImageJ.

### LPS-induced lung inflammation model

Female C57BL/6 mice aged 6–8 weeks were obtained from Charles River Breeding Laboratories (Portage, MI). Different sHDLs were administered i. v. at a dose of 10 mg/kg of 22A, followed by a 10 mg/kg LPS i. p. injection. 18 h post-treatment, the mice were sacrificed. The lung was perfused with 0.5 ml of 10% formalin injected from the trachea before collection.

For *ex vivo* binding assay, the lung tissues were fixed overnight in 10% formalin at room temperature, and switched to 15% then 30% sucrose in PBS for 24 h in each solution at 4°C. The tissues were embedded in optimal cutting temperature (OCT) compound and frozen in isopentane in a liquid nitrogen bath. The tissue sections were sliced using a cryostat and stored at −80°C until use. For *ex vivo* binding studies, sections were thawed at room temperature for 30 min, rehydrated with PBS for 10 min, and blocked with 3% bovine serum albumin (BSA) in PBS with 0.05% Tween-20 (PBST) for 2 h. Then slides were incubated with mVCAM-1 antibody (R&D systems, AF643) in 1% BSA in PBST at 4°C overnight in a humidity chamber. The slides were washed with PBST three times, followed by incubated with an FITC labeled secondary antibody in 1% BSA in PBST (ThermoFisher, #31509) for 2 h at room temperature in dark. The slides were washed with PBST three times again, and incubated with DiD-labeled NT- or VHPK-sHDL for 2 h (22A concentration 10 μg/ml). The slides were washed three times with PBS. DAPI containing mounting media was added to each slide and the slides were sealed with coverslips using clear nail polish. Images were acquired with a Zeiss confocal microscope.

For histological evaluations, the lung was fixed with 10% formalin at room temperature for less than 24 h. The tissues were then embedded in paraffin and sectioned for hematoxylin and eosin (H&E) staining and Ly6G immunohistochemistry (IHC) staining. The numbers of Ly6G^+^ cells in tissue sections were counted using ImageJ. In parallel experiments, blood samples were collected by cardiac puncture at the end of the experiment. Plasma was isolated and stored at −80°C until analysis. The IL-6 and MCP-1 levels were quantified by ELISA (Invitrogen).

### Statistical analysis

Data analysis was conducted using GraphPad PRISM. Statistical significance was determined using a two-tailed unpaired Student’s t-test for two groups of data or a one-way analysis of variance (ANOVA) followed by Tukey test for data of more than two groups. A *p*-value less than 0.05 was considered statistically significant.

## Results

### Preparation and characterization of VHPK-sHDLs

**FIGURE 1:**
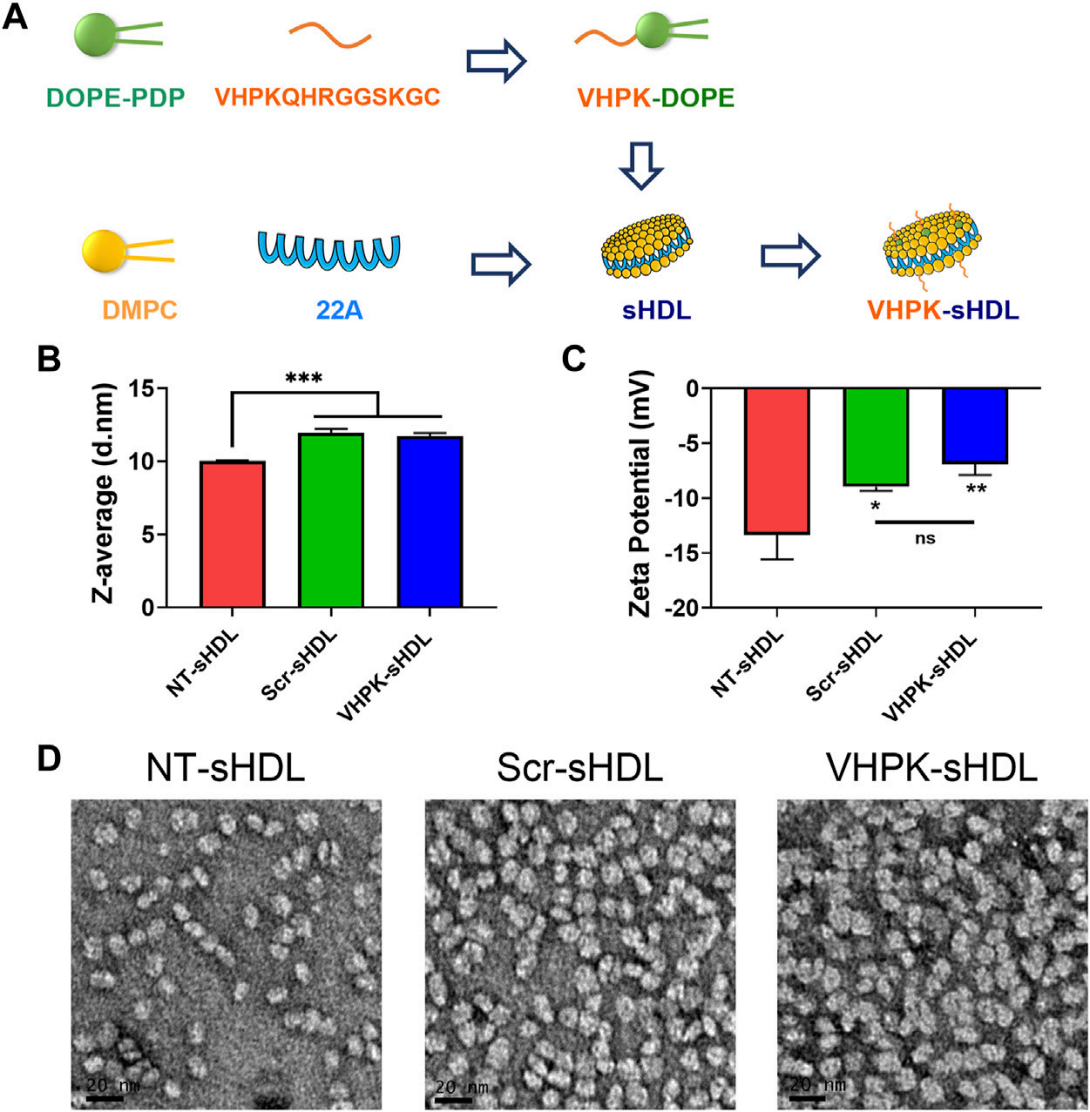
**(A)** Schematic illustration of preparation process of VHPK-sHDLs. Particle size **(B)** and zeta potential **(C)** of different sHDLs measured by DLS (*n* = 3, mean ± SD). **(D)** Representative TEM images of different sHDLs. Scale bar represents 20 μm *p < 0.05, **p < 0.01, ***p < 0.05.

The preparation method for VHPK-sHDLs is illustrated in Figure 1A sHDL nanoparticles composed of ApoA-1 mimetic peptide 22A and DMPC was prepared as described previously (Fawaz et al., 2020). As an amphiphilic peptide (Fawaz et al., 2020), 22A could readily interact with phospholipids such as DMPC, forming disc-like particles with 22A peptide surrounding the rim of a disc of the phospholipid bilayer (Giorgi et al., 2022). No free 22A was detected in prepared sHDLs (Supplementary Figure S1). VHPK peptide DOPE conjugate (VHPK-DOPE) and scrambled peptide DOPE conjugate (Scr-DOPE) were successfully synthesized with a conjugation efficiency of over 90% (Supplementary Figure S2A). By using a post-insertion method, around 90% VHPK-DOPE or Scr-DOPE was inserted into sHDLs by the end of incubation (Supplementary Figure S2B). Insertion of VHPK-DOPE or Scr-DOPE slightly increased the particle size of sHDLs (Figure 1B). As both VHPK and scrambled peptides are positively charged, inserting VHPK- or Scr-DOPE to sHDLs slightly increased the surface charge of conjugated sHDLs (Figure 1C). TEM images showed a uniform particle size distribution of all three sHDL formulations (Figure 1D).

### Cytotoxicity evaluation

**FIGURE 2:**
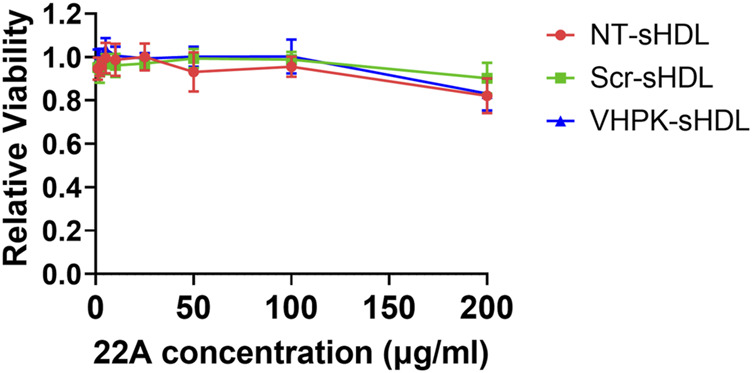
Relative cell viability of HUVEC cells incubated with different sHDLs at indicated concentrations. Cells without treatment were used as 100% (*n* = 6, mean ± SD).

sHDLs have been proved to have a favorable safety profile in previous preclinical and clinical studies (Michael Gibson et al., 2016). As seen in Figure 2, both unconjugated and conjugated sHDLs showed minimal cytotoxicity effects with 22A concentrations as high as 100 μg/ml.

### VCAM-1 dependent endothelial targeting *in vitro*

**FIGURE 3:**
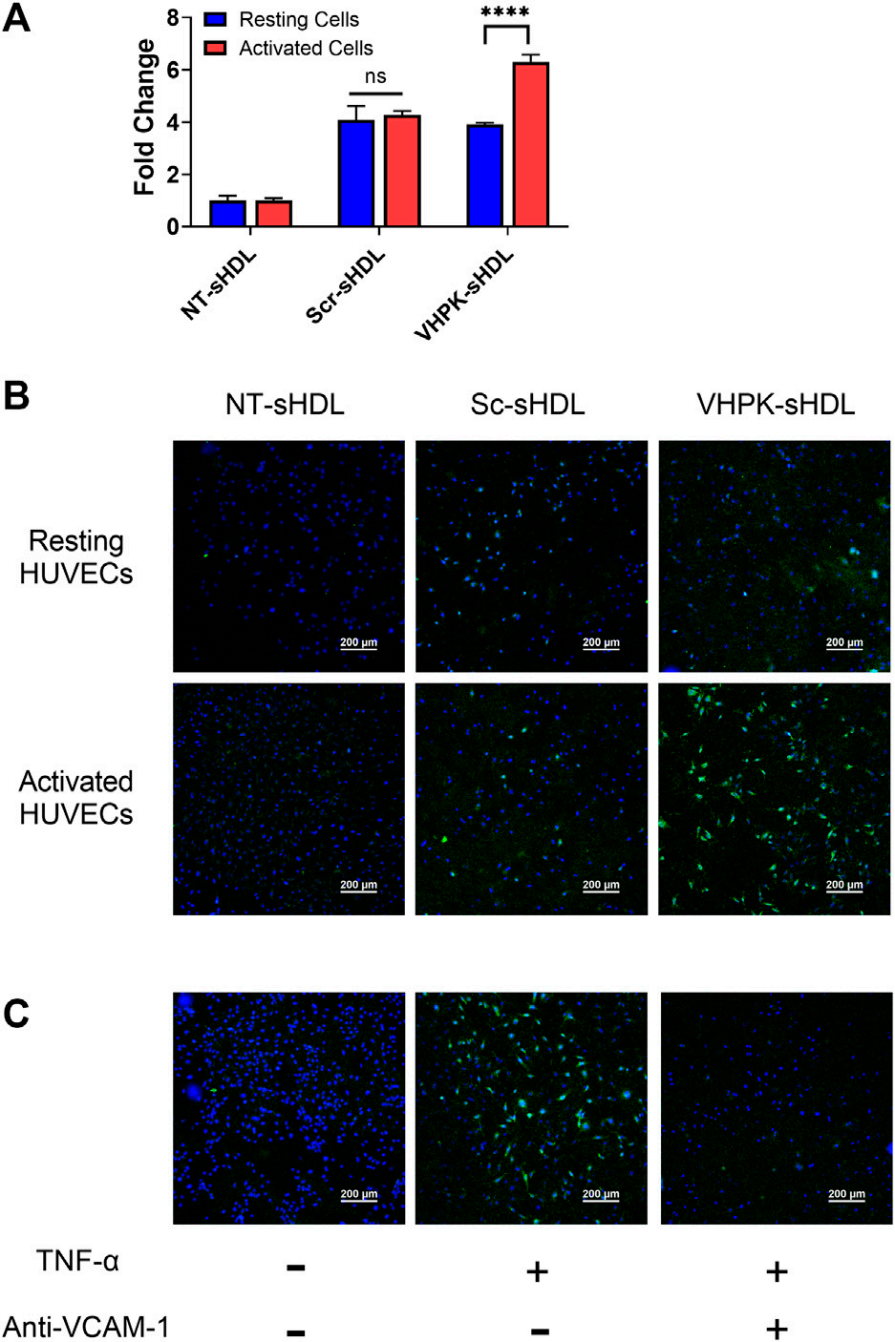
**(A)** Cellular binding of different sHDLs on resting and activated HUVEC cells evaluated by flow cytometry (*n* = 3, mean ± SD. ****p < 0.001). **(B,C)** Representative confocal microscope images of cellular binding of sHDLs on HUVEC monolayers. Blue: Nucleus; Green: DiD-labeled sHDLs.

The cellular binding of different sHDLs was investigated on HUVEC monolayers. HUVEC cells were activated with TNF-α to induce the expression of VCAM-1 (Supplementary Figure S3). As shown in Figures 3A,B, Scr-sHDLs and VHPK-sHDLs showed higher cellular binding on resting HUVEC cells when compared to NT-sHDLs, possibly due to increased non-specific binding caused by the cationic peptide. However, only VHPK-sHDLs showed increased cellular binding on activated HUVEC cells compared to resting cells. Such increased cellular binding was abolished after the binding site of VLA-4 on VCAM-1 was blocked by the pre-incubation with anti-VCAM-1 antibody (Figure 3C), suggesting the enhanced cellular binding of VHPK-sHDLs is mediated by VCAM-1 in the activated HUVECs.

### Cholesterol efflux

**FIGURE 4:**
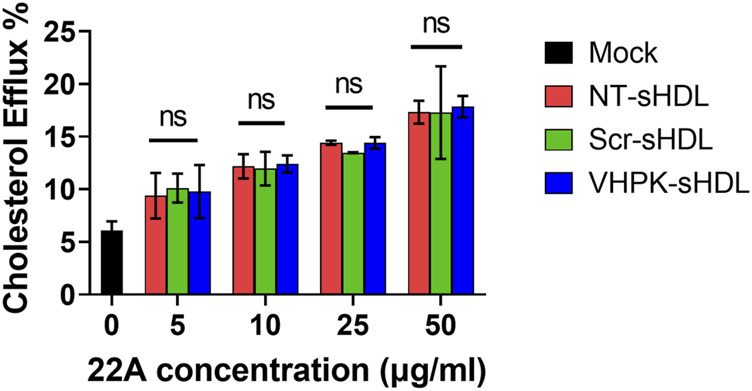
Cholesterol efflux capacity of sHDLs on ^3^H-cholesterol laden J774. A1 cells (*n* = 3, mean ± SD).

As HDL mimetics, sHDLs could efflux cholesterol from peripheral cells (Schwendeman et al., 2015). As shown in Figure 4, a dose-dependent cholesterol efflux effect was confirmed in NT-sHDL, Scr-sHDL, and VHPK-sHDLs. No difference in cholesterol efflux capacity was found in the three kinds of sHDL formulations, suggesting peptide conjugation did not significantly affect the cholesterol efflux capacity of sHDLs.

### Anti-inflammation effects

**FIGURE 5:**
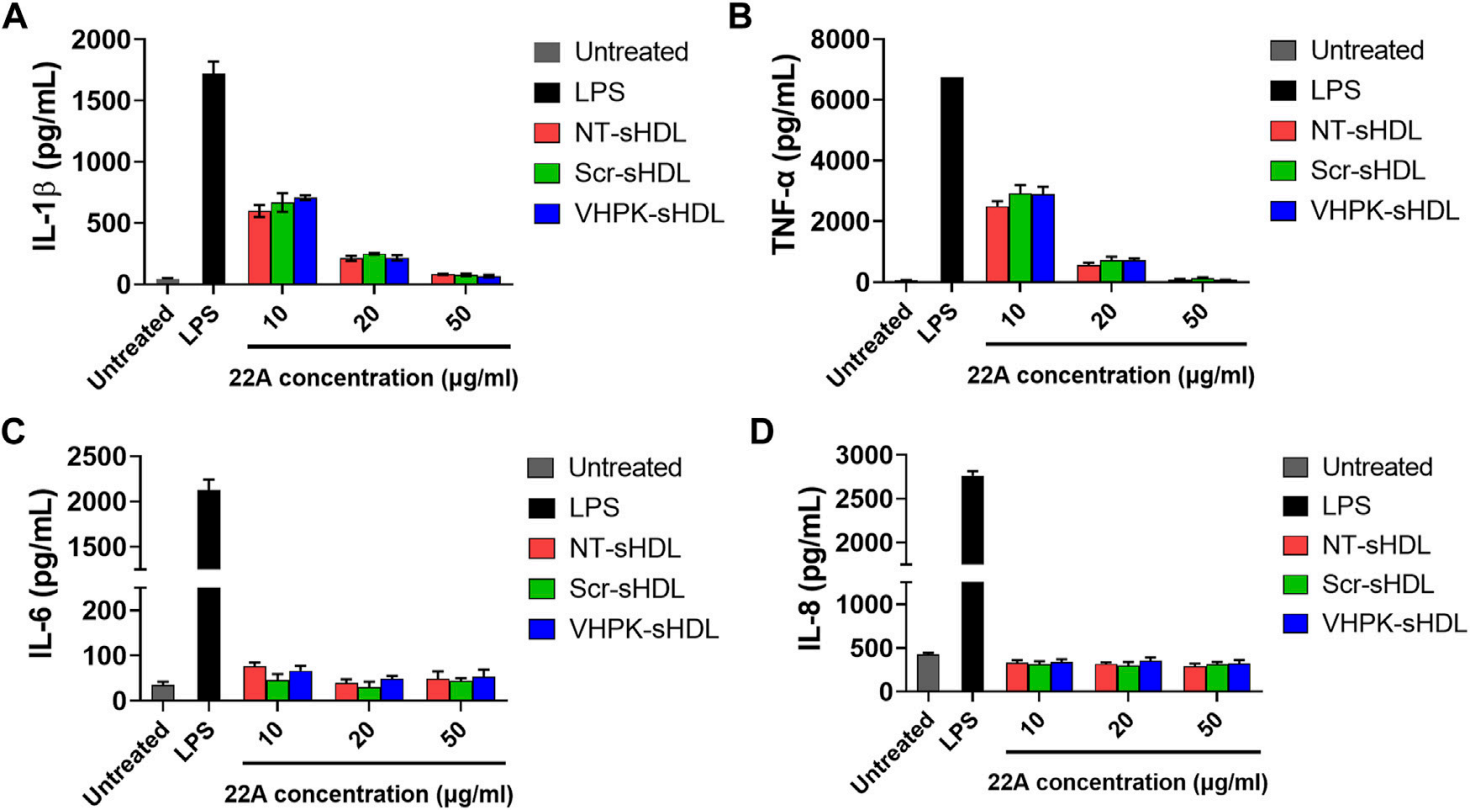
IL-1β **(A)**, TNF-α **(B)**, IL-6 **(C)** and IL-8 **(D)** levels from THP-1 derived macrophages **(A,B)** or HUVECs **(C,D)** after treatment with LPS and different sHDLs. (*n* = 3, mean ± SD).

The anti-inflammatory effects of different sHDLs were examined on HUVECs and THP-1 derived macrophages. As seen in Figure 5, both non-targeted sHDLs and peptide conjugated sHDLs showed potent effects in reducing the production of proinflammatory cytokines induced by LPS, suggesting the introduction of targeting peptides did not affect the anti-inflammatory effects of sHDLs.

### Monocyte adhesion on activated HUVEC monolayers

**FIGURE 6:**
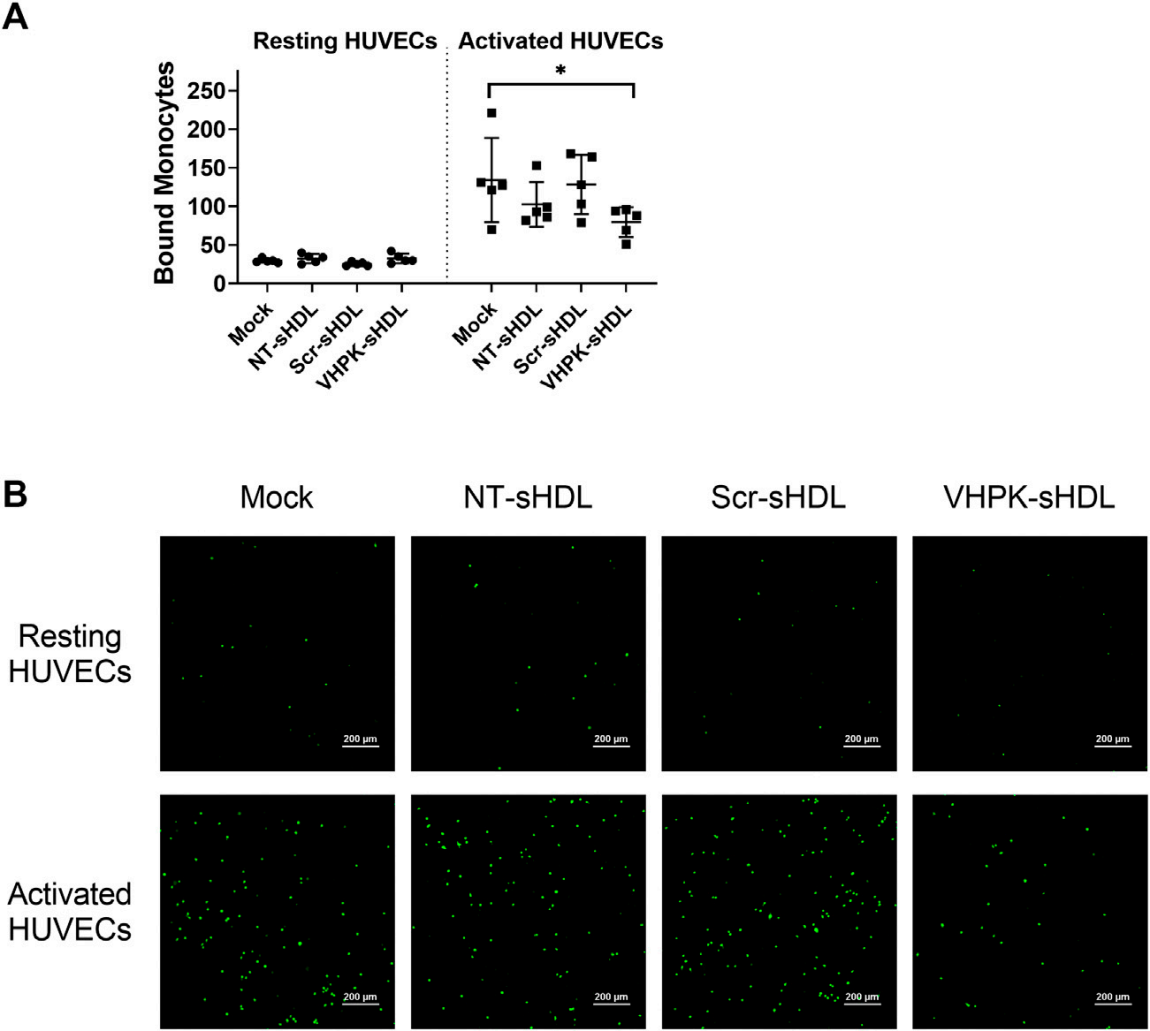
**(A)** Quantification of bound THP-1 monocytes on resting and activated HUVEC monolayers (*n* = 5, mean ± SD, *p < 0.05). **(B)** Representative images of fluorescently labeled THP-1 monocytes adhered on HUVEC monolayers.

As shown in Figure 6, TNF-α activated HUVECs greatly increased the cellular adhesion of THP-1 monocytes. Co-incubating NT-sHDL or Scr-sHDL with monocytes did not affect the cellular adhesion of THP-1 cells. VHPK-sHDLs moderately reduced the adhesion/migration of THP-1 cells on the activated HUVEC monolayer. The lack of total blockage of THP-1 on HUVEC monolayers may be attributed to other adhesion molecules mediating THP-1 adhesion and migration such as ICAM-1 (Medina-Leyte et al., 2020). Interestingly, co-incubating VHPK-peptide with monocytes did not affect the monocyte adhesion on activated HUVEC monolayers (data not shown), suggesting the multivalent binding of VHPK-peptide on sHDLs may be essential for the adhesion reduction effects.

### Inflamed endothelial targeting *in vivo*

**FIGURE 7:**
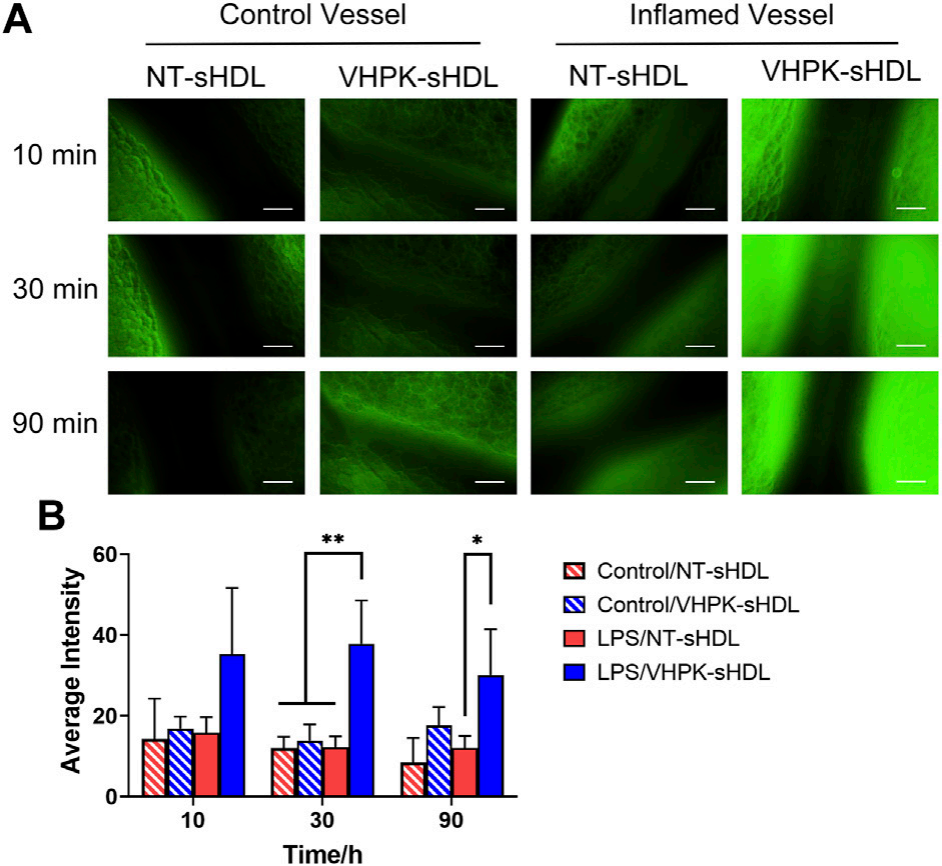
**(A)** Representative intravital microscopy images of the endothelial distribution of NT-sHDLs and VHPK-sHDLs. Scale bar represents 50 μm. **(B)** Average fluorescent intensity on mice endothelium after administration of different sHDLs (*n* = 3, mean ± SD. *p < 0.05, **p < 0.01.).

The endothelial targeting efficiency of sHDLs with or without the targeting peptide was examined *in vivo* using an intravital microscope. LPS was used to induce general vascular inflammation in mice. As seen in Figure 7, following *i. v*. injection, VHPK-sHDLs present an enhanced biodistribution on activated endothelium compared to normal vessels, while the biodistribution of NT-sHDL was similar in normal and inflamed endothelium.

### Therapeutic effects of VHPK-sHDL in LPS-induced inflammation model

**FIGURE 8:**
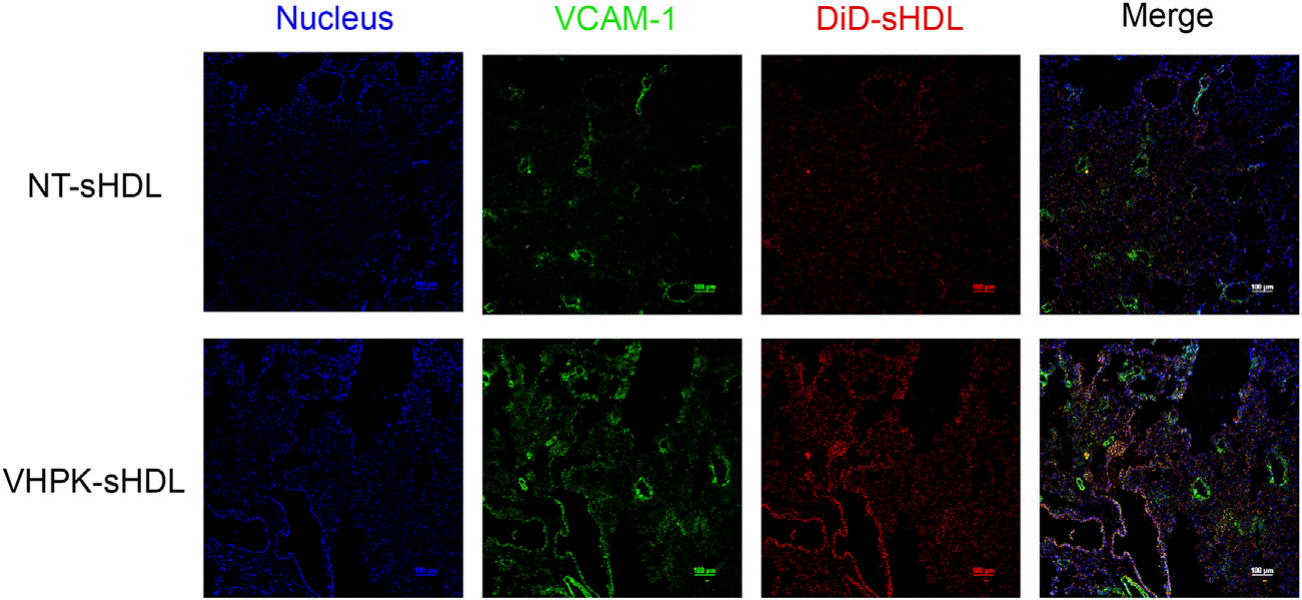
Representative confocal microscopy images showing *ex vivo* binding of NT-sHDL and VHPK-sHDL on the lung tissue sections from LPS-treated mice.

Vascular endothelial inflammation has different manifestations in different inflammatory diseases. For this proof-of-concept study, a high dose of LPS (10 mg/kg) was injected to induce general endothelial inflammation in mice. A biodistribution study was conducted to examine the organ distribution of different sHDLs following systemic injection. Consistent with our previous studies (Schwendeman et al., 2015; Guo et al., 2018; Li et al., 2018; Fawaz et al., 2020), NT-sHDL and VHPK-sHDLs showed high accumulation in the liver, the primary elimination organ for HDL, as well as organs with large endothelium areas such as lung and kidney (Supplementary Figure S5). VHPK-sHDL showed higher signals compared to NT-sHDL, which may be attributed to both increased unspecific binding and active targeting to inflamed endothelium in the liver following systemic LPS injection (Supplementary Figure S5). The lung was chosen as the organ of interest due to its vast endothelium area and well-characterized endothelial dysfunctions including high VCAM-1 expression levels following the LPS challenge (Supplementary Figure S4). The *ex-vivo* binding assay showed that compared to NT-sHDLs, VHPK-sHDLs presented a higher particle binding as well as a higher co-localization with VCAM-1 on lung tissue sections (Figure 8), which is consistent with the *in vitro* findings.

**FIGURE 9:**
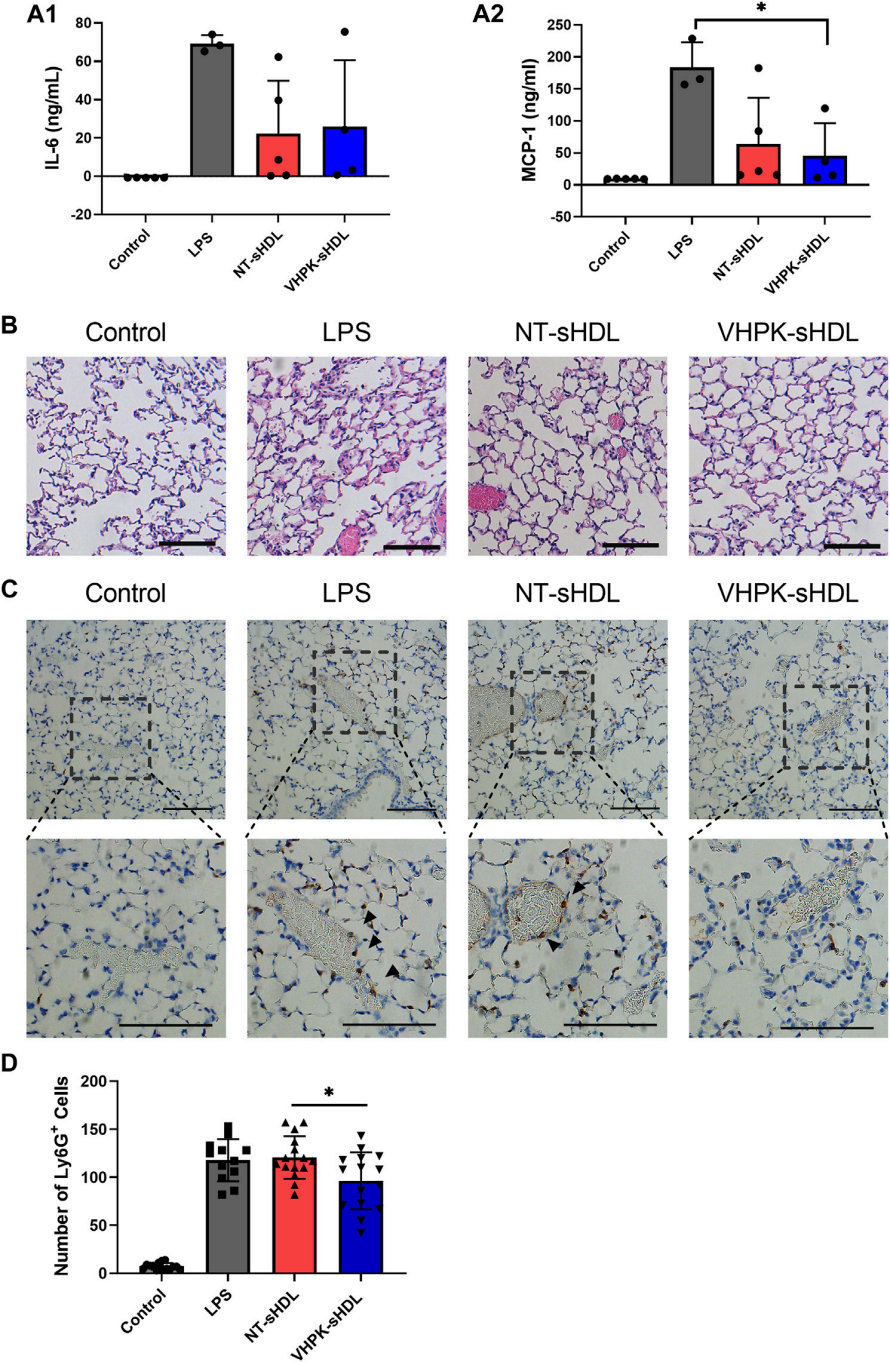
Plasma IL-6 **(A1)** and MCP-1 **(A2)** levels in mice 18 h after administration of LPS and sHDLs. (n = three to five, mean ± SD, *p* < 0.05). **(B)** Representative H&E staining of lung tissue sections of mice treated with different sHDLs. **(C)** Representative images of Ly6G IHC staining of lung sections mice treated with different sHDLs. Control indicates mice without LPS or sHDL treatment. Arrows indicate endothelial bound Ly6G^+^ cells. The scale bar represents 100 μm. **(D)** Numbers of Ly6G^+^ cells counted from IHC stained lung tissue sections (*n* = 12-15, mean ± SD. Ly6G^+^ cells were counted from three random 20x fields from slides of each mouse).

As shown in Figure 9B, while lung tissues from LPS-treated mice presented significant tissue damages manifested by interalveolar septal thickening and interstitial edema, the tissue injury was less significant in NT-sHDL or VHPK-sHDL treated groups. The proinflammatory cytokine levels in plasma were quantified with ELISA. A large intra-group variance was observed, suggesting significant individual differences in LPS tolerance and responses to treatment. Mice treated with VHPK-sHDLs presented lower plasma MCP-1 levels and showed a trend for lower IL-6 levels in plasma (Figure 9A). Ly6G^+^ leukocytes, which are major species of cells to infiltrate in lung inflammation, were stained by immunohistochemistry (IHC). As shown in Figure 9C, fewer Ly6G^+^ cells were found infiltrated lung tissues in VHPK-sHDL treated mice than that in NT-sHDL treated groups. While the difference between Ly6G^+^ counts in VHPK-sHDL treated group and the LPS-treated group was not statistically significant (*p* = 0.06), IHC analysis showed fewer Ly6G^+^ cells were found bound on the endothelium in VHPK-sHDL treated group, implying the potential for VHPK-sHDL to inhibit leukocyte adhesion and infiltration to inflamed tissues.

## Discussion

As the interface between systemic circulation and sites of inflammation, vascular endothelium is an essential targeting site for drug delivery in inflammatory diseases. Moreover, inflamed endothelium plays important roles in disease initiation and progression through recruiting leukocytes into inflamed tissue and producing inflammation mediators, making it a critical therapeutic target in inflammatory diseases. Thus, inflamed endothelial cells are both appealing delivery targets and potential therapeutic targets for the diagnosis and treatment of inflammatory diseases. Among various biomarkers on inflamed endothelium, VCAM-1 has been one of the most frequently studied targeting sites for activated endothelium. Numerous VCAM-1 targeted delivery systems have been developed to deliver imaging agents, small molecule drugs, and mRNAs to inflamed endothelial cells (Ailuno et al., 2020). While most of the VCAM-1 targeting delivery systems were designed for the diagnosis and treatment of atherosclerosis (Thayse et al., 2020), the applications have been broadened to other inflammatory diseases. For example, Garello et al. developed a VCAM-1 targeted paramagnetic micelles for neuroinflammation imaging (Garello et al., 2018). In another study, a VLA-4 decorated cell membrane-coated nanoparticle was used to deliver dexamethasone to inflamed lungs (Park et al., 2021).

With HDL-mimicking components and structure, sHDLs have been shown to have multiple HDL-like functions, including cholesterol efflux, anti-inflammatory, and endothelial protective functions (Kuai et al., 2016; Thaxton et al., 2016). Typical HDL-mimicking particles are formulated with the full-length ApoA-1 protein, which is costly and challenging for large-scale manufacturing (Caparon et al., 2010). In this study, a more scalable and cost-efficient 22-mer ApoA-1 mimetic peptide, namely 22A, was used to formulate sHDLs. Previous studies suggested that 22A-containing sHDLs have a strong neutralization capacity against endotoxins (Guo et al., 2022). Through dislocating TLR-4 from cholesterol-rich lipid rafts, 22A containing sHDLs could also suppress TLR-4 signaling pathways and inhibit the production of proinflammatory cytokines from macrophages and endothelial cells (Kim, 2019; Guo et al., 2022). One of the 22A-containing sHDLs, ETC-642, has entered clinical trials and showed good safety profile and cholesterol mobilization capacity after a single dose intravenous administration (Miles et al., 2004; Di Bartolo et al., 2011). However, sHDLs offer little tissue specificity after systemic administration. For example, in a clinical study on atherosclerosis patients, sHDL showed only ∼10% more biodistribution in inflamed plaque regions compared to normal arterial tissues (Zheng et al., 2016). Enabling an active targeting of sHDLs to inflamed endothelium may be an effective strategy to increase the delivery efficiency of sHDLs to target tissues.

In the present study, a VCAM-1 targeting VHPK peptide was conjugated to the surface of sHDLs to enable an active targeting to inflamed endothelial cells. *In vitro* cellular binding study showed that VHPK-sHDLs have increased cellular binding on activated HUVEC cells compared to resting cells in a VCAM-1 dependent manner. The cellular binding, instead of cellular uptake, was used to evaluate the delivery efficiency of sHDLs in the present study due to the unique interaction mechanisms of HDL and HDL mimetic particles with endothelial cells. While endothelial cells are capable of endocytosis and transcytosis of HDLs, the component exchange between endothelial cells and HDL can occur on the cell surface without particle internalization. For example, endothelial cells have been found to selectively uptake HDL-associated components such as cholesterol, vitamin E, or hydrophobic dye through surface receptors without uptaking the entire HDL particle (Goti et al., 2001; Plochberger et al., 2018). Thus, cellular binding might be an important indicator of delivery efficiency of sHDLs. The increased delivery efficiency of VHPK-sHDLs to inflamed endothelium was also observed *in vivo* in the intravital microscopy study.

A series of *in vitro* studies were conducted to investigate whether VHPK-sHDLs preserve the anti-inflammatory effects of non-targeted sHDLs. Results showed that VHPK-sHDLs present comparable cholesterol efflux and anti-inflammatory effects to NT-sHDL, suggesting that VHPK peptide did not negatively affect the protective function of sHDLs. Interestingly, VHPK-sHDL reduced the monocyte adhesion on the activated HUVEC monolayers, which might be attributed to the competition between VLA-4 expressing monocytes and VHPK-sHDLs on VCAM-1 binding. Similar results were also observed in another VCAM-1 targeted nanoparticle (Kelly et al., 2005), suggesting additional therapeutic mechanisms introduced by the conjugation of VCAM-1 targeted peptide.

Inflammation has diverse manifestations in different inflammatory diseases. For example, vascular inflammation in atherosclerosis is hallmarked by oxidized lipoproteins, monocyte infiltration, and cholesterol-laden foam cells in the subendothelial space (Gimbrone and Garcia-Cardena, 2016). In acute lung injury, uncontrolled inflammation is characterized by excessive production of proinflammatory cytokines as well as neutrophil infiltration (Maniatis et al., 2008). While it is beyond the scope of this study to examine the efficacies of VHPK-sHDLs in every kind of inflammatory disease, as a proof-of-concept study, the therapeutic effects of VHPK-sHDLs were evaluated using an LPS-induced inflammation model. A sublethal dose of LPS was given to mice through i. p. injection. In addition to evaluating systemic inflammation levels by serum cytokine levels, the lung was chosen as an organ of interest to evaluate the efficacy of sHDLs due to its vast endothelium surface area and well-characterized endothelial dysfunctions. Consistent with the previous study, both non-targeted and targeted sHDLs showed the potential to reduce inflammatory cytokine levels in plasma, which could be attributed to the LPS neutralization and anti-inflammation capacities of sHDLs (Guo et al., 2022). When focusing on the lung tissues, H&E staining showed alleviated lung injury in NT- and VHPK-sHDL treated mice. Moreover, mice with VHPK-sHDL treatment showed less infiltration of Ly6G^+^ leukocytes, which may suggest enhanced effects of VHPK-sHDLs on inhibiting leukocyte recruitment. It is worth noting several limitations of the i. p. LPS induced general inflammation model. First, the i. p. injection of a sublethal dose of LPS led to a large variance of inflammatory responses in mice. Second, the systemic administration of LPS complicated the efficacy analysis in lung tissues, as both systemic and local inflammatory response contributes to the results. Thus, while the present animal study provided proof-of-concept results on the therapeutic potential of VHPK-sHDL, a more defined animal model will be used to optimize VHPK-sHDL for specific inflammatory diseases.

## Conclusion

VCAM-1 specific, inflamed endothelial targeted VHPK-sHDLs were prepared in the present study. The active targeting of VHPK-sHDLs to inflamed endothelial cells was demonstrated by *in vitro* and *in vivo* results. Conjugation of VCAM-1 targeting ligand did not compromise the cholesterol efflux and anti-inflammatory effects of sHDLs, and may provide additional protective effects by inhibiting leukocyte adhesion to activated endothelium. Based on the results of this proof-of-concept study, VHPK-sHDLs hold the potential to be further optimized to fully exert therapeutic potential to inflammatory disease either as a stand-alone therapy or drug delivery carrier.

## Data Availability

The raw data supporting the conclusion of this article will be made available by the authors, without undue reservation.
