# Supplementary material for: Development of activated endothelial targeted high-density lipoprotein nanoparticles
Source: Front Pharmacol. 2022 Aug 29;13:902269. doi: 10.3389/fphar.2022.902269 (PMC9464908; doi:10.3389/fphar.2022.902269)
Supplement: Supplementary file 1 [file DataSheet1.PDF]

Supplementary Materials

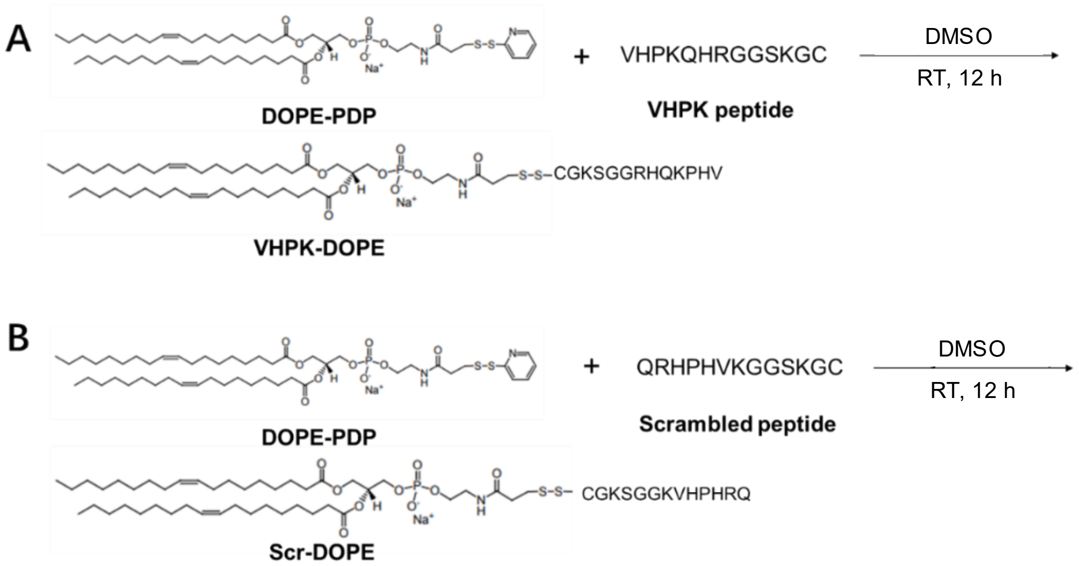

Scheme S1. Synthesis of VHPK-DOPE (A) and Scr-DOPE (B) conjugates.

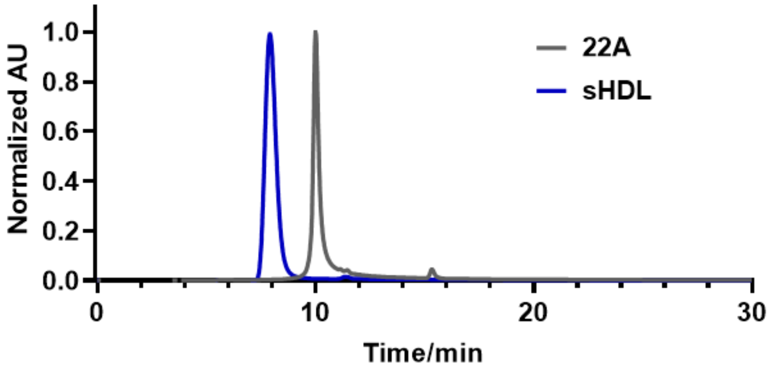

Figure S1. Gel permeation chromatography of free 22A peptide and sHDLs.

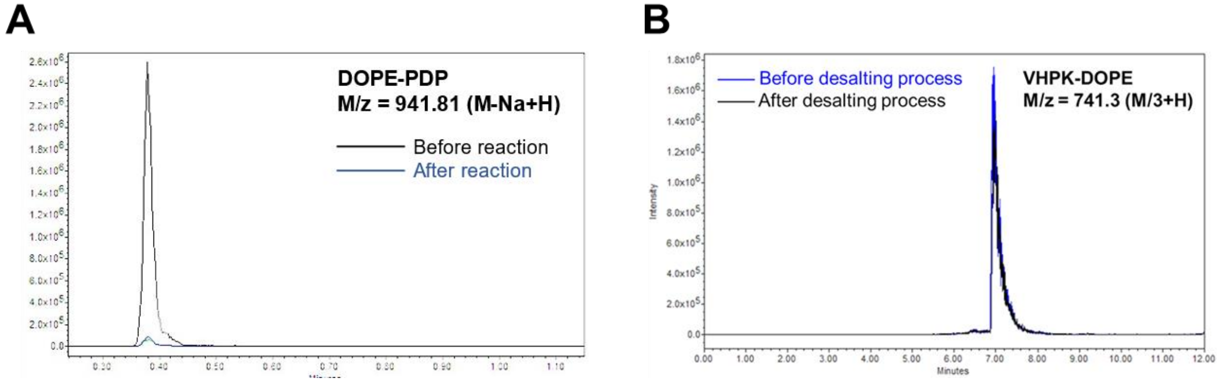

Figure S2. LC/MS spectrum showing (A) quantification of unreacted DOPE-PDP before and after conjugation reaction, and (B) quantification of VHPK-DOPE before and after removing uninserted VHPK-DOPE using desalting columns.

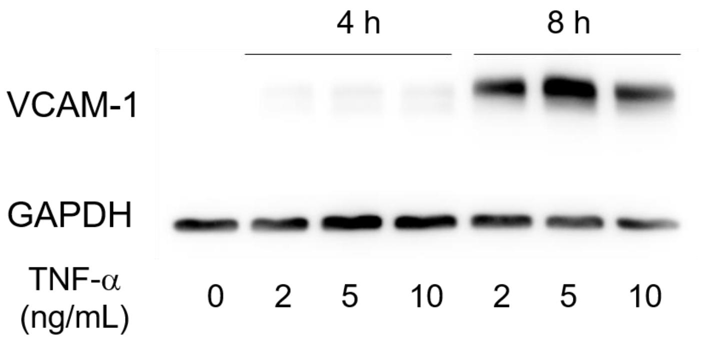

Figure S3. VCAM-1 expression on HUVEC cells at different time points after TNF-α activation with different concentrations.

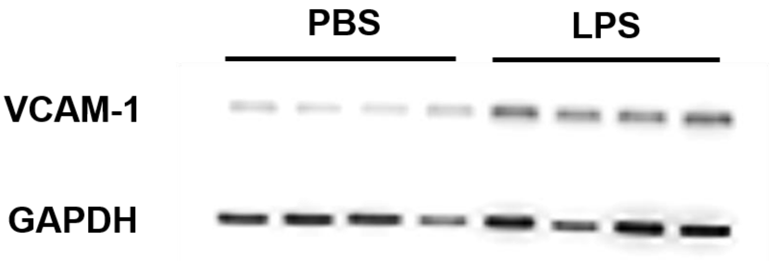

Figure S4. VCAM-1 expression in mice lung tissues 18 h post administration of PBS or LPS (10 mg/kg) (N = 4 per group).

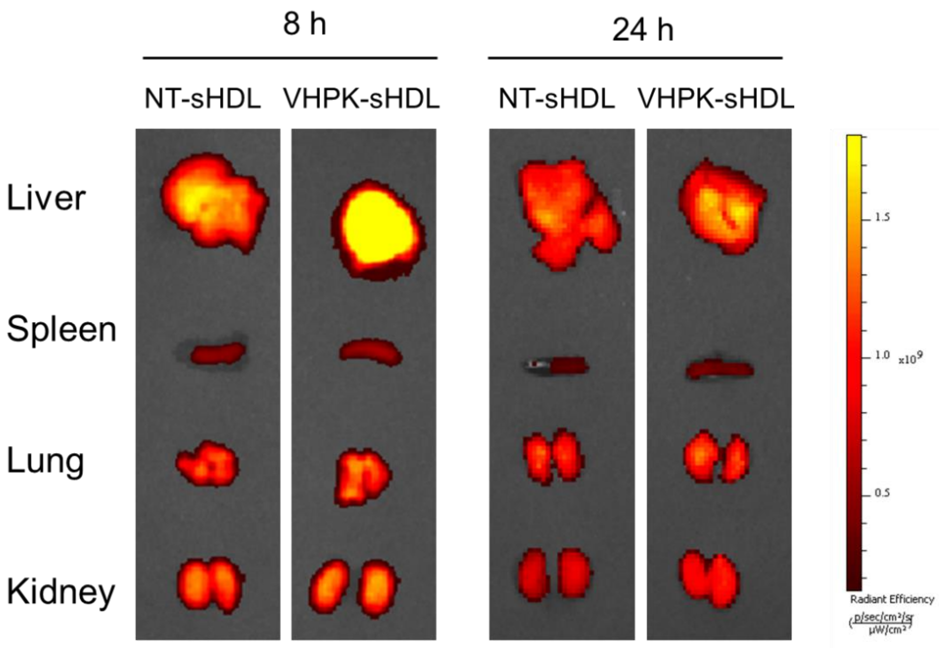

Figure S5. Biodistribution of different sHDLs in major organs following *i.v.* injection to LPS-treated mice. Female C57BL/6 mice aged 6-8 weeks were obtained from Charles River Breeding Laboratories (Portage, MI). Different DiR-labeled sHDLs were administered *i.v.* at a dose of 10 mg/kg of 22A, followed by a 10 mg/kg LPS *i.p.* injection. Different time points post-injection, the mice were sacrificed. major organs were extracted and imaged using IVIS.
